# Improvement in Impaired Social Cognition but Not Seizures by Everolimus in a Child with Tuberous Sclerosis-Associated Autism through Increased Serum Antioxidant Proteins and Oxidant/Antioxidant Status

**DOI:** 10.1155/2019/2070619

**Published:** 2019-11-23

**Authors:** Kunio Yui, George Imataka, Hitomi Sasaki, Yohei Kawasaki, Tohru Okanshi, Ryoichi Shiroki, Shigemi Yoshihara

**Affiliations:** ^1^Department of Urology, Fujita Health University School of Medicine, Aichi 470-1192, Japan; ^2^Department of Pediatrics, Dokkyo Medical University, Mibu, Tochigi 321-0293, Japan; ^3^Clinical Research Center, Chiba University Hospital, Chiba 260-8677, Japan; ^4^Department of Child Neurology, Seirei Hamamatsu General Hospital, Hamamatsu 430-8558, Japan

## Abstract

We investigated the effect of the mammalian target of rapamycin (mTOR) inhibitor everolimus on tuberous sclerosis complex- (TSC-) associated autistic symptoms and focal seizures with impaired awareness in a female child with TSC. We further evaluated the relationship between improved autistic symptoms and seizures and increased the serum levels of the antioxidant proteins, ceruloplasmin (Cp) and transferrin (Tf), and oxidant-antioxidant status indicated by the oxidant marker oxidized low-density lipoprotein (ox-LDL) and the antioxidant marker total antioxidant power (TAP). Everolimus treatment improved impaired social cognition and autistic behaviors; however, seizure and epileptic activity persisted. Serum Cp and Tf levels gradually increased in response to improved autistic symptoms. Serum TAP levels gradually decreased from baseline to the lowest value at 16 weeks and then increased at 24 weeks, showing a trend toward decreased total score of the Aberrant Behavior Checklist. This study revealed that everolimus treatment improved impaired social cognition with increased serum levels of the copper mediator (Cp) and iron mediator (Tf) via homeostatic control of mTOR activity accompanied by overlap of the oxidant-antioxidant system. Everolimus had no effect on TSC-related epileptiform discharges, and thus, the autistic symptoms and epileptic activity may be two independent end results of a common central nervous system disorder including mTOR hyperactivity. This trial is registered with JMAS-IIA00258.

## 1. Introduction

Tuberous sclerosis complex (TSC) is a rare multisystem monogenic hamartomatous disorder [1] with a high prevalence of epilepsy and neuropsychiatric symptoms [2, 3]. Accumulating evidence has highlighted high comorbidity rates of autism spectrum disorder (ASD) and impaired social cognition in patients with TSC [4, 5]. However, few case reports have described ASD-associated impairments in social cognition in patients with TSC. Social cognition refers to the cognitive function of information processing, interpretation of socioemotional information in others [6], and cognitive processes such as recognition, accurate processing, and the effective use of social cues [7].

TSC is caused by a mutation in either the tuberous sclerosis complex 1 (*TSC1*) or complex 2 (*TSC2*) genes [1]. A heterozygous mutation results in the hyperactivation of the mammalian target of rapamycin (mTOR), inducing tumorous symptoms and autistic symptoms. These symptoms can be inhibited by mTOR inhibitors such as rapamycin [3, 8]. Indeed, several previous studies have suggested that mTOR inhibitors improved deficient social interactions and abnormal autistic behaviors in patients with TSC [9]. However, few studies have investigated the therapeutic efficacy of the mTOR inhibitor everolimus on impaired social cognition. Although mutations of *TSC1* or *TSC2* induce mTOR hyperactivity, how this mechanism contributes to the development of ASD remains unclear [2, 10]. Interestingly, animal models with heterozygous mutations in *TSC1* or *TSC2* exhibit ASD-like social deficits in the absence of cortical lesions [10], suggesting that other neurobiological mechanisms may contribute to the development of autistic symptoms. The mTOR pathway is closely related to oxidative stress [11] and antioxidant capacity [12]. Oxidative stress-induced reactive oxygen species enhance mTOR activity [11], whereas antioxidant effects block the mTOR signaling pathway [13]. In particular, iron-related transferrin (Tf) [14] has been implicated in mTOR activation. Tf is the main protein involved in the delivery of iron to the brain.

The abnormal accumulation of iron in the brain contributes to neurodegenerative processes; thus, Tf is believed to have an important role in the regulation of brain iron homeostasis [15]. Tf uptake modulates the mTOR signaling pathway [16] via tristetraprolin [14], which regulates cellular signaling [17] to reduce the toxic effects of iron accumulation and to promote cell growth [16]. In addition, ceruloplasmin (Cp) is the primary copper-binding protein [18] with an essential role in regulating copper and iron homeostasis to prevent the formation of free radicals [19]. Moreover, Cp is involved in determining the rate of iron efflux from cells with iron stores [20]. The accumulation of iron or decreased Cp activity in the brain has been associated with neurodegeneration [19]. Thus, Tf and Cp play essential roles in the development of neurodegenerative diseases, including TSC [21].

Several studies have investigated the relationship between Cp and mTOR pathway alterations. We previously described how everolimus improved both social impairment and repetitive behaviors, and these improvements were accompanied by increases in the serum levels of both Cp and Tf [22]. Copper inhibits mTOR pathway-activated autophagy [23]. Furthermore, oxidized low-density lipoprotein (ox-LDL) is an oxidative stress marker [24], and inhibition of reactive oxygen species (ROS) production may reduce autophagy to suppress ox-LDL-induced platelet activation by activating the PI3K/AKT/mTOR pathway [25], indicating that ox-LDL inhibits mTOR activity. Collectively, everolimus treatment may attenuate the upregulated mTOR activity accompanying increased serum Cp and Tf levels and alterations in the oxidant-antioxidant system. To investigate the oxidant-antioxidant status, serum levels of ox-LDL and total antioxidant power (TAP) were measured [26].

As mTOR regulates neuronal excitability in already established neural circuits, mTOR hyperactivation enhances neural excitability related to seizures [27], inducing epileptiform discharges (referred to as spikes) in the electroencephalogram (EEG) and may contribute to progressive brain dysfunction, including autistic symptoms [28]. However, the association between epileptic encephalopathy and ASD remains open to debate [28].

In this study, we demonstrate the therapeutic potential of the mTOR inhibitor everolimus in the amelioration of ASD-associated impaired social cognition and behavioral symptoms accompanied by increased serum levels of Tf and Cp, which were partially consistent with changes in the oxidant-antioxidant system, in a young girl with TSC. The present findings shed further light on novel mechanisms underlying TSC-associated autistic symptoms. This study further examined the association between epileptiform discharges and TSC-related autistic symptoms.

## 2. Case

### 2.1. Technical Information

ASD was diagnosed by one psychiatrist and one pediatrician who specialized in ASD using the DSM-5, Autism Diagnostic Interview-Revised (ADI-R), and the Autism Diagnostic Observation Schedule (ADOS). The ADI-R was usually used as a diagnostic instrument, but the ADOS was useful for studying longitudinal changes in core autistic symptom severity. The serum levels of Tf, Cp, ox-LDL, and TAP were assayed at baseline and 4, 8, 12, 16, and 24 weeks after the initiation of everolimus treatment. Serum VEGF-D levels were assessed to examine the patient's response to everolimus at baseline and 4, 8, 12, 16, and 24 weeks after the initiation of everolimus treatment. Serum everolimus levels were measured at 12, 16, and 24 weeks after the initiation of the treatment. Social cognition was assessed using the Social Communication Questionnaire (SCQ) and the Social Responsiveness Scale (SRS) [29]. Behavioral symptoms were assessed using the Aberrant Behavior Checklist (ABC).

### 2.2. Patient Clinical Characteristics

The patient was an eight-year-old girl with TSC that was accompanied by the core social and behavioral symptoms of ASD and focal seizures with impaired awareness. At the age of four months, the patient exhibited a focal seizure with impaired awareness, which was characterized by clusters of epileptic spasms from the right face to the left hand clonic seizure without hypsarrhythmia, following language development delay. She was treated with anticonvulsants such as sodium valproate, which reduced the frequency of her seizures. The patient continued to take anticonvulsants. At the age of two years, she experienced recurrence of her partial seizure. Anticonvulsant therapy reduced her seizure frequency; however, her seizures sometimes recurred. Her refractory seizures resulted in her complete corpus callosotomy.

**Figure 1:**
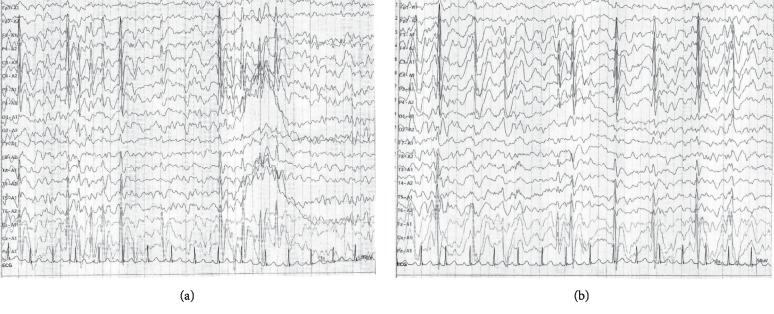
Electroencephalography before everolimus treatment (a) and after everolimus treatment (b).

At seven years of age, her EEG recording indicated high-voltage irregular slow waves intermixed with spikes and polyspikes, which are sometimes observed in patients with focal seizures characterized by impaired awareness [30] that were detected bilaterally in the frontal and central areas (Figure 1).

### 2.3. Ultrasound Examination and Magnetic Resonance Imaging (MRI) Findings

**Figure 2:**
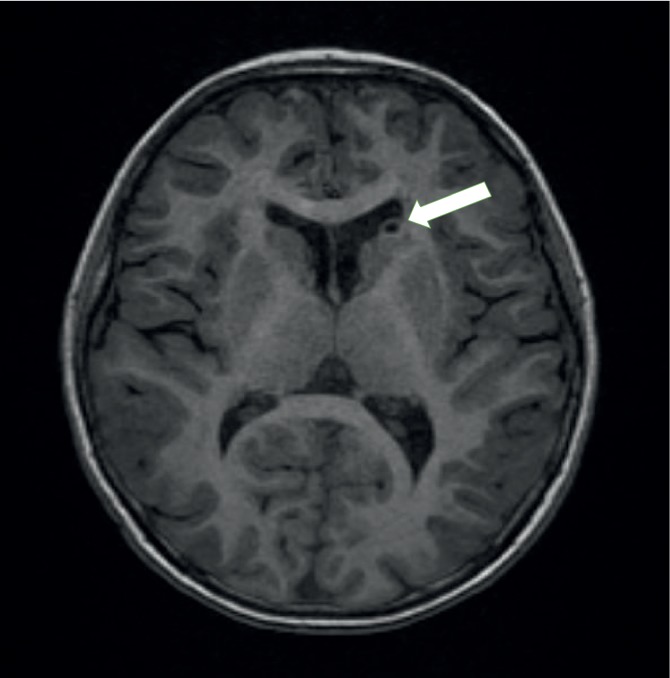
MRI finding of a subependymal nodule (SEN) (white arrow).

At three years of age, abdominal ultrasonography revealed several small angiomyolipomas (AMLs) in both kidneys. At seven years of age, MRI revealed a subependymal nodule (SEN) (Figure 2). These findings, including the SEN and AML, met the diagnostic criteria of TSC [31]. Thus, a TSC diagnosis was confirmed without genomic analysis.

### 2.4. Autistic Symptoms

At six years of age, the patient gradually developed impairment of social cognition, which primarily included impaired social interaction and communication. She sometimes spoke broken Japanese in a faltering manner. She did not respond to various forms of nonverbal communication with the typical facial expressions or physical gestures. She was unable to understand the interests and needs of other children. The patient was often observed to be engaged in “parallel play” at the edge of a group. In addition, she did not engage in pretend play. Although she exhibited a desire to form friendships with other children, she often demonstrated inappropriate friendliness and a lack of awareness of other children's interests and needs because she was unable to understand these factors. Therefore, it was difficult for her to form and sustain interactions with other children, thus resulting in a withdrawal into repetitive play and behaviors. The patient was unable to understand what her teachers and classmates said; thus, her teachers sometimes had to provide guidance on what to expect from others. She sometimes lost her temper when things did not go the way she wanted. These clinical features indicated deficits in social cognitive processes, such as an impairment in understanding the mental state of others [32, 33], impaired interpretation of socioemotional information [6], and deficits in the effective use of social cues [7].

The patient displayed a continuous acquisition of motor abilities but with stagnated performance. Her play tended to have a persistent sensorimotor or ritualistic quality, and her behaviors were characterized by repetitive and stereotyped patterns of activities, such as repeated finger sucking. She exhibited a delayed acquisition of motor skills and had difficulty with motor coordination, postural control, and imitation of the movements of other children.

Collectively, the patient displayed autistic symptoms, including impaired social cognitive function and impaired social communication. Her behavioral symptoms consisted of repetitive and stereotyped behaviors.

### 2.5. Psychometric Assessment of Autistic Symptom

At seven years of age, she was diagnosed by one psychiatrist and one pediatrician who specialized in ASD using the DSM-5, ADI-R, and ADOS. As shown in Table 1, her ADI-R scores were above the autism diagnostic cutoff scores for qualitative abnormalities in reciprocal interaction (21; cutoff = 10), qualitative abnormalities in communication for both the verbal and nonverbal total scores (19; cutoff = 15), and repetitive/stereotyped patterns of behavior (3; cutoff = 3). These ADI-R scores confirmed the diagnosis of mild ASD.

**Figure 3:**
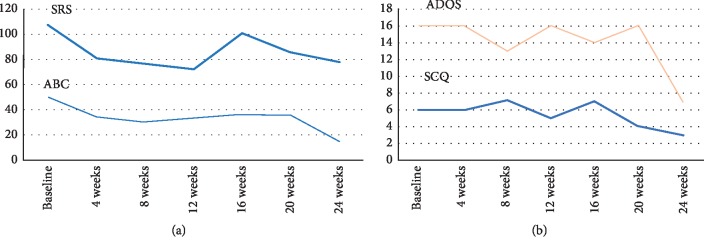
Changes of total scores of (a) SRS and ABC and (b) SCQ and ADOS during everolimus treatment.

As the social cognition domain is related to information processing about others [6] and the effective use of social cues [7], parental reports of communication and reciprocal social skills such as the SRS [29] and SCQ [29, 34] have been used to assess social cognition [3]. These SRS and SCQ subscales assess social awareness, social information processing, capacity for reciprocal social communication, social motivation, and repetitive/restricted interests [29]. The SCQ is a useful tool for screening ASD and to determine whether further assessment is needed in children with suspected ASD [35]; however, it is more difficult to detect changes in the severity of ASD using the SCQ. Therefore, we used the SCQ to detect ASD symptoms in this TSC patient with autistic symptoms. The autistic behavioral symptoms were assessed using the ABC. The ABC is intended to evaluate treatment responses in psychopharmacological and behavioral intervention trials for children and adolescents with normal intelligence quotient (IQ) levels [36].  Figure 3 shows the total scores on the SRS, ABC, SCQ, and ADOS. The patient had a total score of 16.0 on the ADOS module 2 algorithm, which was greater than the average total ADOS score (8.94 ± 9.71) reported in 33 adolescents with high-functioning ASD [37]. Her SRS total scores (107) were comparable to the scores for patients with ASD, and the total ABC score (50) was greater than the scores of 29 ASD individuals with an age range of 13–27 years (total SRS and ABC scores were 120.21 and 60.14, respectively) [38]. Because total SRS scores greater than 76 indicate severe levels of social reciprocity difficulties [39], the patient was considered to have impaired social interaction. In addition, because total SCQ scores above 17 indicate social communication difficulties [40], the patient's SCQ score of 6 was within the normal range. Collectively, the patient was considered to have impaired social reciprocity but not social communication. At six years of age, she underwent an intelligence test using the Wechsler Intelligence Scale (WISC)-5, and her total IQ was 75, indicating normal levels of intellectual functioning [41]. Other evaluations revealed no additional abnormalities.

### 2.6. Biochemical Measurement

Serum vascular endothelial growth factor-D (VEGF-D) is a useful biomarker of TSC-associated lymphangioleiomyomatosis and can be used in therapeutic decision making [42]. Importantly, serum VEGF-D may be useful for monitoring responses to treatment strategies using mTOR inhibitors, including changes in the kidney AML size [43] and TSC severity [44] in patients with TSC. The inhibition of phospho-mTOR significantly decreased tumor cell VEGF-D levels *in vitro*, indicating the existence of the Akt/mTOR-VEGF-C/VEGF-D axis [45]. Therefore, to examine the patient's response to everolimus, serum VEGF-D levels were assessed in this study. As described above, the copper mediator Cp [46] and iron mediator Tf [14] are known to be associated with mTOR activity.

Furthermore, ox-LDL suppresses the P13K/AKT/mTOR signaling pathway [25]. Therefore, the serum levels of Cp, Tf, ox-LDL, and TAP were measured at baseline and 4, 8, 12, 16, 20, and 24 weeks after initiation of everolimus treatment.

## 3. Results

The patient's total scores on the ABC as well as the SRS gradually decreased during treatment, indicating an improvement in autistic symptoms (Figure 3). In particular, when comparing scores at baseline to those at the end of the 24-week treatment, her SRS total score decreased from 107 before treatment to 78 (a 27.2% decrease) and her SCQ total score decreased from 6 before treatment to 3 (a 50% decrease) (Table 2). An approximate 20% reduction in the SRS total score may be considered a response to treatment [47]. Thus, the patient's cognitive profiles, including impaired social cognition, also improved. Thus, treatment with everolimus induced improvements in impaired social cognition.

**Figure 4:**
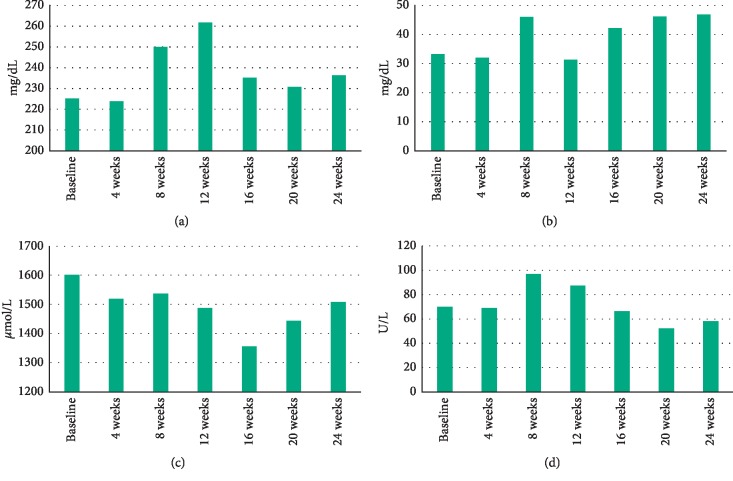
Changes of serum levels of (a) Tf, (b) Cp, (c) TAP, and (d) ox-LDL.

As shown in the bar graph in Figure 4, serum Tf and Cp levels gradually increased from baseline to 8 and 12 weeks, in accordance with symptom improvement as indicated by decreased total scores on the ABC as well as the SRS. Serum ox-LDL levels gradually increased from baseline to the peak value at 8 weeks and then gradually decreased. Serum TAP levels gradually decreased from baseline to their lowest value at 16 weeks of treatment and then gradually increased. Thus, there appeared to be a positive yet nonsignificant relationship between Tf and ox-LDL levels in serum. The total scores on the SRS, ABC, ADOS, and SCQ tended to increase at 8–12 weeks after everolimus treatment; plasma Tf and ox-LDL levels also increased with a trend toward decreased total scores on the SRS and ABC (Figures 3 and 4).

The dosage of everolimus was 2.5 mg/day during the 24-week treatment. Serum everolimus levels during the maintenance dosing period were 9.88, 6.37, and 22.10 ng/mL at 16, 20, and 24 weeks after initiation of treatment, respectively. The patient's serum levels of VEGF-D were 505.8 mg/mL at baseline, 657.0 at 4 weeks, 698.7 ng/mL at 8 weeks, and 567.0 ng/mL at 12 weeks and gradually increased to 777.9 ng/mL at 16 weeks, 769.3 mg/mL at 20 weeks, and 1119.7 pg/mL at 24 weeks after initiation of treatment. No severe or life-threatening side effects related to the everolimus treatment were observed.

Her several small AMLs disappeared; however, the size of the SEN did not change after the 24-week everolimus treatment.

Antiepileptic medications, including lamotrigine and sodium valproate, did not have any observable therapeutic effects in the patient. The frequency of seizures did not significantly change after treatment with everolimus at a dose of 2.5 mg. Everolimus treatment improved impaired social interaction with others, repetitive finger sucking, and ability to comprehend others' intentions, and her feelings increased as she exhibited an effective response to her mother's scolding. However, her seizures and epileptiform discharges persisted. Thus, at nine years of age, she received a complete corpus callosotomy, which eliminated the seizures and epileptiform discharges. She was able to enjoy time with her classmates in school. These findings indicated that 2.5 mg/day of everolimus improved social functioning. However, seizure and epileptiform discharges persisted.

## 4. Discussion

Social cognition is involved in multiple forms of information processing, including sensitivity to various social signals, facial expressions, and eye gaze directions [48]. The patient exhibited deficits in social communication areas, including poor eye contact, failure to respond to others, poor attention related to impairment of understanding other's mental status [32], and impairment of social communication, including gestures and shared enjoyment. These clinical features are consistent with those previously reported in TSC patients with impaired social cognition [5, 6, 32, 48].

The present study indicated that treatment of a girl with TSC with everolimus for 24 weeks increased the serum levels of antioxidant proteins such as Tf as well as Cp, gradually improved ASD-related social impairment, such as social cognition and social withdrawal, and reduced autistic behaviors including repetitive behavior. However, seizures and epileptiform discharges persisted.

Serum everolimus levels were 9.88, 6.37, and 22.2 ng/mL at weeks 16, 20, and 24 after initiation of treatment, respectively. A previous study found that serum everolimus levels in the range of 3–8 ng/mL were associated with good efficacy and safety profiles [49]; thus, serum everolimus levels during everolimus treatment in this patient may have been appropriate for improving social and behavioral impairments. The patient's serum levels of VEGF-D gradually increased in response to improvements in autistic symptoms, indicating a response to everolimus treatment.

Total SRS scores decreased by 27.2%. A previous randomized, double-blind, parallel-group study reported that the antidepressant sertraline induced a >25% decrease in the Hamilton Depression Rating Scale (HAMD) scores and was significantly more effective than control drugs [50]. Another randomized, double-blind, parallel-group study reported that the antidepressant sertraline induced a >25% decrease in the placebo-controlled study, indicating that a 25% decrease in the assessment scale was an improvement [51]. Moreover, a 50% reduction from the initial score was considered a clinically significant improvement [52]. Thus, a 27.2% decrease in the total SRS score and a 50% decrease in the total SCQ score indicated that everolimus treatment significantly improved social impairment, including social cognition.

Impairment of social cognition was evident approximately six years after the onset of her seizures. These findings may be comparable to those in a previous research article, which indicated that cognitive difficulties as indicated by IQ were evident four years after the onset of seizures [53]. As described above, the everolimus dose was 2.5 mg/day, which did not eliminate her focal seizures with impaired awareness. However, her social functioning gradually improved. Several review articles have suggested a close association between cognitive function and epileptic encephalopathy in patients with TSC [3, 28]. However, whether improved seizure control results in secondary improvements in social cognition remains open to debate [3, 28]. It is important to note that TSC-related autistic symptoms and TSC-related epileptiform discharges may have been independent consequences of brain dysfunction in this patient.

Serum VEGF levels gradually increased from baseline (505.8 pg/mL) to a peak value (1119.7 pg/mL) at week 24. A previous study found that the average serum level of VEGF in 19 healthy children was 306.1 ± 39.4 pg/mL [54]. The baseline serum VEGF-D level in this patient was higher than in healthy children [54]. As serum VEGF-D levels have been considered a biomarker of the response to treatment with an mTOR inhibitor [43, 44], gradual increases in the serum VEGF-D levels indicated the response to everolimus.

The SEN size was not changed after the 24-week treatment with everolimus. Although several previous studies have found a 50% reduction in the subependymal giant cell astrocytoma (SEGA) size, other studies have shown only slight decreases in the SEGA size after everolimus treatment [10, 55]. A previous study indicated that everolimus treatment did not change the histopathological characteristics of the SEGA in a 15-year-old girl [55]. Moreover, the effect of everolimus was not easily characterized using TSC lesions such as SEGA and AML [55]. No reduction in the SEN size observed in this patient may thus be reasonable. Previous studies have demonstrated that everolimus treatment may prevent the development of AML and SEGA [56]; therefore, other neurobiological mechanisms may contribute to the beneficial effect of everolimus treatment on autistic symptoms.

VEGF-D deficiency may result in oxidative stress [57]; exogenous stimulation with VEGF-D induces an antioxidant response in human endothelial cells [58]. Moreover, everolimus attenuated oxidative stress by altering antioxidant capacity [12] and/or reversing the accumulation of oxidative stress-related ROS [59]. Notably, everolimus treatment gradually elevated serum VEGF-D levels during the course of everolimus treatment. Therefore, everolimus treatment may contribute to increased serum levels of antioxidants such as serum Cp and Tf via elevated serum VEGF-D levels. Cp is the main copper-binding protein in blood plasma [18] and an important serum antioxidant [60]. Increased levels of intracellular copper suppressed mTOR activation [61], and copper treatment downregulated mTOR signaling [62]. In addition, the mTOR signaling pathway modulated Tf uptake [16] and iron homeostasis through the Tf receptor [14]. Moreover, the Tf receptor can be used to measure intracellular changes in mTOR activity [63]. Collectively, our present findings suggest that everolimus may have increased cellular antioxidant capacity by enhancing serum Cp and Tf levels. Both Cp and Tf regulate the transfer of iron, with the activity of Cp modulated by Tf [64]. These findings indicated a close association between Cp and Tf. Although the patient exhibited increased Cp levels during the 8–24 weeks of treatment, her serum Cp levels (26–47 mg/dL) were within the normal limits or higher value according to the SRL information (reference levels, 21–37 mg/mL) (http://testguide.srl.info/hachioji/test/detail/011732702). The patient did not exhibit any general symptoms of copper toxicity, such as burning stomach pain, nausea, vomiting, diarrhea, jaundice, hair loss, anemia, anorexia, anxiety, attention deficit disorder, arthritis, or asthma [65]. Thus, increased serum levels of Cp were not related to copper toxicity. Although elevated serum Tf levels are a clinical sign of malignant lymphomas [66], the patient never exhibited symptoms of this disease. Tf saturation reflects iron availability [67]. Tf levels gradually increased from 250 mg/dL at week 8 to a peak value of 262 mg/dL at week 12 and remained at higher values (231–235 mg/mL) than the baseline value (225 mg/mL) (Figure 4). The Tf levels were higher than the reference range (200–340 mg/mL) based on the SRL information database (http://testguide.srl.info/hachioji/test/detail/011741302). Thus, this patient may or may not have had an iron deficiency. Collectively, a gradual increase in serum Tf and Cp levels appeared to be a response to everolimus treatment. As described above, evidence from previous studies supports the present finding that everolimus attenuates mTOR hyperactivity by increasing both serum Cp and Tf levels.

Oxidized low-density lipoprotein (ox-LDL) suppresses the P13K/AKT/mTOR signaling pathway [25]. The mTOR pathway is closely related to oxidative stress [11] and antioxidant capacity [12]. Indeed, there appears to be a positive yet nonsignificant relationship between Tf and ox-LDL levels in plasma (Figure 4). Clinical evidence suggests that plasma ox-LDL levels are significantly associated with plasma antioxidant markers such as glutathione peroxidase [68]. This is in agreement with the hypothesis that a positive association between ox-LDL and glutathione peroxidase activity might reflect an adaptive mechanism to prevent further oxidative imbalance in the face of high ox-LDL concentrations [68]. However, another review article reported no significant association between LDL and total antioxidant capacity [69]. These previous studies support our seemingly overlapping curves for Tf and ox-LDL as well as the relationships between plasma Tf and ox-LDL levels. The total scores on the SRS, ABC, ADOS, and SCQ tended to increase at 8–12 weeks after everolimus treatment in response to increased plasma TAP and ox-LDL levels (Figure 4). Thus, antioxidant properties may partially overlap with the aforementioned homeostatic mechanisms.

## 5. Conclusion

The current study indicated that everolimus treatment for 24 weeks increased serum levels of antioxidant proteins, including Cp and Tf, inducing a gradual improvement in impaired social cognition and repetitive behavior in a female child with TSC. However, everolimus treatment did not eliminate her seizure or epileptiform discharges. The present findings suggested that epileptiform activity and autistic symptoms may be independent consequences of brain dysfunction related to mTOR hyperactivation.

## Figures and Tables

**Table 1:** Clinical characteristics of the case.

Age	Female, 8 years
Age at onset of ASD	6 years
MRI or echo	(i) AML on bilateral kidneys
	(ii) SEN left on the anterior horn of lateral ventricle
ASD features	(i) Impaired communication
	(ii) Wrapping up doll play with repeated finger-sucking.
ADI-R score	ADI-R
	Reciprocal interaction: 21
	Communication: 19
	Repetitive behaviors: 13
Total scores of SRS and ABC	SRS: 50
	ABC: 107
WISC IQ	67
Everolimus doses	2.5 mg/day for 24 weeks
Results	ABC score: 50% decrease
	SRS score: 26% decrease
	Social response and repetitive behaviors were improved
Serum levels of Tf and Cp	Serum Cp and Tf levels increased from baseline to 24 weeks of treatment in accordance with symptom improvement
Serum levels of TP, ox-LDL, and creatine	Serum TAP levels showed trend toward opposite decreased ABC scores
	Serum creatine levels showed no definite alteration

AML: angiomyolipomas; SEN: subependymal nodule; ADI-R; Autism Diagnostic Interview-Revised; ABC: Aberrant Behavior Checklist; SRS: Social Responsiveness Scale; Cp: ceruloplasmin; Tf: transferrin; TAP: total antioxidant power; ox-LDL: oxidized low-density lipoprotein.

**Table 2:** Changes of ADI-R, ADOS, ABC, and SRS total scores between baseline and 24 weeks after everolimus treatment.

Variable	Baseline	24 weeks	% decrease
ADI-R total			
ADI-R social interaction domain	21	14	33.3
ADI-R communication domain	19	10	47.4
ADI-R restricted/repetitive behavior domain	3	0	100.0
ADOS	16	7	56.2
ABC	50	15	70.0
SRS	107	78	27.2
SCQ	6	3	50.0

ADI-R: Autism Diagnostic Interview-Revised; ADOS: Autism Diagnostic Observation Schedule; ABC: Aberrant Behavior Checklist; SRS: Social Responsiveness Scale; SCQ: Social Communication Questionnaire. % decrease = scores at 24 weeks − the baseline scores/baseline scores × 100.
